# Design and Fabrication of a 3D-Printed Microfluidic Immunoarray for Ultrasensitive Multiplexed Protein Detection

**DOI:** 10.3390/mi14122187

**Published:** 2023-11-30

**Authors:** Keshani Hiniduma, Ketki S. Bhalerao, Peyahandi I. Thilini De Silva, Tianqi Chen, James F. Rusling

**Affiliations:** 1 Department of Chemistry, University of Connecticut, Storrs, CT 06269-3060, USA; keshani.hiniduma@uconn.edu (K.H.); ketki.bhalerao@uconn.edu (K.S.B.); peyahandi.de_silva@uconn.edu (P.I.T.D.S.); tqchen@stanford.edu (T.C.); 2 Institute of Materials Science, University of Connecticut, Storrs, CT 06269-3136, USA; 3 Department of Surgery and Neag Cancer Center, Uconn Health, Farmington, CT 06030-0001, USA; 4 School of Chemistry, National University of Ireland at Galway, H91 TK33 Galway, Ireland

**Keywords:** design and fabrication, 3D printed, microfluidic, protein detection, cancer, multiplex

## Abstract

Microfluidic technology has revolutionized device fabrication by merging principles of fluid dynamics with technologies from chemistry, physics, biology, material science, and microelectronics. Microfluidic systems manipulate small volumes of fluids to perform automated tasks with applications ranging from chemical syntheses to biomedical diagnostics. The advent of low-cost 3D printers has revolutionized the development of microfluidic systems. For measuring molecules, 3D printing offers cost-effective, time, and ease-of-designing benefits. In this paper, we present a comprehensive tutorial for design, optimization, and validation for creating a 3D-printed microfluidic immunoarray for ultrasensitive detection of multiple protein biomarkers. The target is the development of a point of care array to determine five protein biomarkers for aggressive cancers. The design phase involves defining dimensions of microchannels, reagent chambers, detection wells, and optimizing parameters and detection methods. In this study, the physical design of the array underwent multiple iterations to optimize key features, such as developing open detection wells for uniform signal distribution and a flap for covering wells during the assay. Then, full signal optimization for sensitivity and limit of detection (LOD) was performed, and calibration plots were generated to assess linear dynamic ranges and LODs. Varying characteristics among biomarkers highlighted the need for tailored assay conditions. Spike-recovery studies confirmed the assay’s accuracy. Overall, this paper showcases the methodology, rigor, and innovation involved in designing a 3D-printed microfluidic immunoarray. Optimized parameters, calibration equations, and sensitivity and accuracy data contribute valuable metrics for future applications in biomarker analyses.

## 1. Introduction

Microfluidics systems development incorporates principles and technologies from numerous fields, including engineering, chemistry, physics, biology, material science, fluid dynamics, and microelectronics. Microfluidic systems manipulate and control small volumes of fluids within flow networks or single microchannels to accomplish a target task in a nearly automated fashion. Volumes used in these systems vary from microliters to femtoliters. In biotechnology and medical diagnostics, microfluidics can provide researchers a tool to access a broad range of experiments and assays with high precision, sensitivity, and speed using minimal amounts of sample and reagents [1,2]

Microfluidics offers significant advantages for a wide variety of devices, including microreactors for synthesizing pharmaceuticals and manufacturing microelectronics [3,4,5]. Devices can integrate multiple functions into ‘lab-on-a-chip’ (LOC) configurations, with applications ranging from enhancing analytical methodologies to addressing critical challenges in healthcare, environmental monitoring, and biological research [6,7]. These LOC devices are capable of transporting individual micro-samples within the microfluidic environment without the need for continuous fluid flow to open avenues for highly sensitive and specific analyses [8]. The versatility of LOC platforms is underscored by their integration with various detection schemes. Some devices employ plasmonic sensing techniques, leveraging the interaction of light with nanostructured metals to enhance sensitivity in detecting biomolecules [9]. LOC also have the potential to reshape the landscape of environmental monitoring [10]. They can achieve single-cell analysis and address genomics, proteomics, and drug screening [11,12,13,14]. Related ‘organs-on-a-chip’ are microfluidic devices designed to model functions of human organs in vitro for the study of disease mechanisms and drug toxicity [4,15,16].

Microfluidic immunoarrays are another example of microfluidic devices. They have been used for applications such as disease diagnosis and drug development [17]. Some devices use microfluidics to help detect biological molecules, such as glucose or cholesterol. Sometimes known as “microfluidic biosensors”, they have been used for applications such as monitoring blood glucose in diabetic patients and detecting food-borne pathogens in food [18,19,20]. Microfluidic devices, especially those utilizing 3D printing, can be designed for decentralized and point-of-care use, such as hospitals, clinics, or homes, not requiring costly equipment or specialized personnel [13,15,21].

Our research team has developed 3D-printed immunoarrays for clinical diagnostics for the detection mainly of proteins in biological fluids as biomarkers for cancers [21,22,23,24,25] and COVID-19 [26]. Microfluidic systems have been designed to detect analytes, including hormones, enzymes, drugs, and disease-specific biomarkers [27,28]. The significance of 3D-printed immunoarrays in clinical diagnostics lies in their sensitivity, specificity, and versatility. They can be designed to detect low concentrations of analytes with high precision and accuracy. In addition, assay designs can facilitate fast and easy protocols, requiring only small amounts of sample and minimal equipment [19,20,21,22,23,24,25,26,27,28,29,30].

Cost-effective production of complex microfluidic designs was previously challenging using traditional fabrication methods [31]. While 3D printers cannot yet match the nm size resolutions of some lithographic methods, low cost, rapid design, and turnaround are appropriate for multiplexed immunoassays. The ability to quickly correct design errors has sped up development of 3D-printed microfluidic devices and made it possible to rapidly create customized and optimized systems [22,32]. The process of 3D printing has enabled researchers with limited resources to access microfluidic technology to contribute new innovations [13].

In this paper, we present a comprehensive tutorial on design and development of a 3D-printed microfluidic array for ultrasensitive chemiluminescence (CL) detection of multiple proteins in a single droplet of blood or serum. We address conceptualizing and designing a microfluidic immunoarray and adaptation to a specific application. We then outline the progression to generate a 3D digital model of the microfluidic immunoarray. To facilitate this process, tutorials available on the AUTODESK website and YouTube can be referenced, providing a structured approach to learning the necessary Fusion 360 software [33]. The design process encompasses the incorporation of features such as detection wells, microchannels, reservoirs, and other elements into the array, ensuring the realization of a robust immunoassay. We specifically describe the utilization of a Formlabs Form 2 3D printer to produce a design characterized by the precision and resolution necessary for multiple protein detection. We highlight strategies to attain desired performance and optimized analytical capabilities for an ultrasensitive immunoassay. The ultimate aim is to present a cost-effective (<$1 per assay), user-friendly device with minimal training requirements for use, with excellent selectivity, and fast ultrasensitive detection of multiple proteins.

## 2. Materials and Methods

### 2.1. Materials

All reagents and chemicals were analytical grade. Chitosan (low molecular weight, 448869), glutaraldehyde (G5882), and calf serum (C8056) were from Sigma Aldrich (St. Louis, MO, USA). Blocker Casein in PBS buffer (37528) was from Thermo Fisher Scientific (Waltham, MA, USA). DuoSet ELISA kits for Human Pigment Epithelium-Derived Factor (PEDF) (DY1177), Human Cancer Antigen 125 (CA-125) (DCA125), Human Interleukin-8 (IL-8) (DY208), Human Cluster of Differentiation 14 (CD-14) (DC140), and Human Cysteine-rich angiogenic inducer 61 (Cyr61) (DCYR10) were purchased from R&D Systems (Minneapolis, MN, USA). Bovine serum albumin (BSA) (5217) was from Tocris Bioscience (Bristol, UK). Streptavidin-Poly (Horseradish Peroxidase) (Poly-HRP80, 65R-S118) conjugate was obtained from Fitzgerald^®^ (Gardner, MA, USA). Chemiluminescence (CL) was generated using Thermo Fisher (Rockford, IL, USA) SuperSignal^®^ West Femto Maximum Sensitivity Substrate (34095), containing femto-luminol and hydrogen peroxide mixed immediately before use. Pooled human serum was purchased from Innovative™ Research (Novi, MI, USA). The superglue used to stick the flap to cover the detection wells on the 3D-printed chip was from Loctite^®^ (Westlake, OH, USA). CL was measured using a Syngene^®^ (Frederick, MD, USA) dark box with a CCD camera. Images were processed using GeneSnap^®^ (version 7.09.17) and GeneTools software (version 4.01.04). Phosphate buffer saline (PBS) pH 7.4 was prepared in lab as 0.01 M sodium phosphate in 0.14 M NaCl and 2.7 mM KCl. Phosphate buffer saline-tween 20 (PBS-T20) was prepared as 0.1% Tween-20 in phosphate buffer saline (PBS) pH 7.4. A Form 3 3D printer was purchased from Formlabs (Somerville, MA, USA), and Clear Resin (RS-F2-GPCL-04) from the same company was used for printing. A programmable syringe pump (model Fusin Fusion 400) was obtained from Chemyx (Stafford, TX, USA).

### 2.2. Methods

The construction of the 3D-printed microfluidic immunoarray is performed in 3 steps. First, the immunoarray is designed and 3D printed. Conducting the immunoassay and assay validation are the 2nd and the 3rd steps. Within each step, there are intermediate optimization steps as well. The following is a detailed explanation of each of the above steps.

#### 2.2.1. Designing and 3D Printing the Array

**Figure 1:**
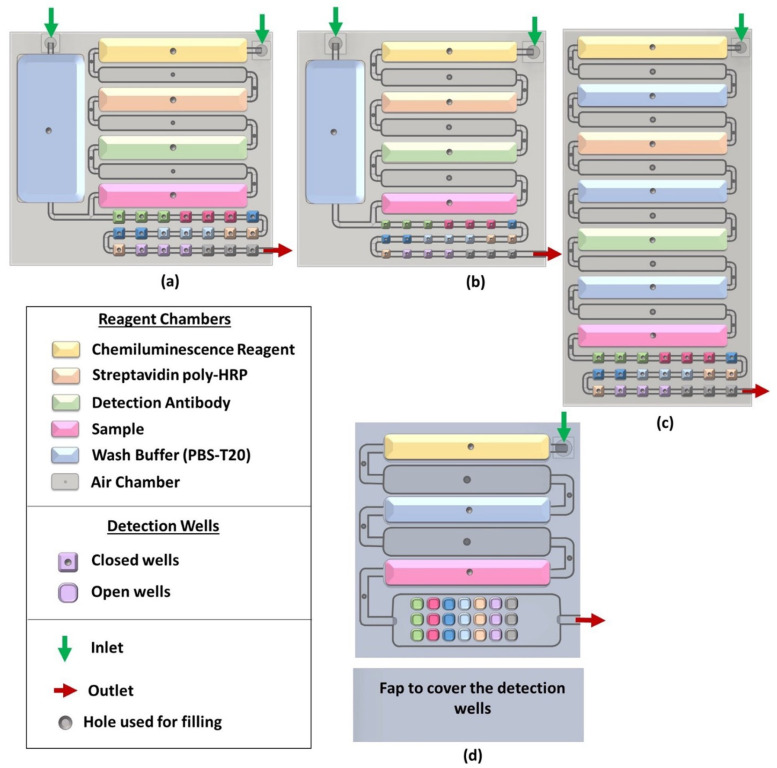
Evolution of the 3D-printed devices: (**a**) version 1 with 10 µL detection wells and the wash buffer reservoir on the side, (**b**) version 2 with 5 µL detection wells and the wash buffer reservoir on the side, (**c**) version 3 with the wash buffer chamber included with the rest of the reagent chambers, and (**d**) version 4/final version with open detection wells.

The microfluidic device is designed using Fusion 360 software based on predetermined design parameters, which are microchannel dimensions, shape, configuration of the microchannels and the associated functionalization areas (reagent chambers and detection wells), multiplexed detection method, number of reagents needed to perform the assay, material, and printer used for 3D printing. The dimensions of the design are subjected to changes depending on how suitable the 3D-printed design is to perform the intended sandwich immunoassay (Figure 1). First, a 2D sketch of the microfluidic device footprint is drawn in the Fusion 360 software. Then, using the ‘extrude’ feature in the software, this sketch is converted into a 3D object. Additional features such as microchannels, reagent chambers, detection wells, micropump connectors, and holes are then added. These features are added inside the 3D object created above by making the sketch on a plane inside it and then ‘extruding’ it to form a cavity inside. Another way is to break the design into layers, construct them separately, and then assemble them as one device. Then, the prototype arrays are 3D printed.

Next, the prototype microfluidic device is subjected to flow dynamic tests by using a programmable micropump and colored solutions. Colored solutions are added into the chambers to check whether they hold the intended solution volumes and whether solutions flow smoothly and without mixing. Colored solutions also help to detect flow blockages and unwanted solution mixing. This can be supplemented by using computational fluid dynamic (CFD) software [34]. The flow rate and the dimensions of the additional features are readjusted so that there is no mixing of reagents, no pressure drops, and no immobilized molecules removed by the flow, e.g., antibodies removed from the detection wells. This can be checked by monitoring a response from detection wells for a single analyte.

**Figure 2:**
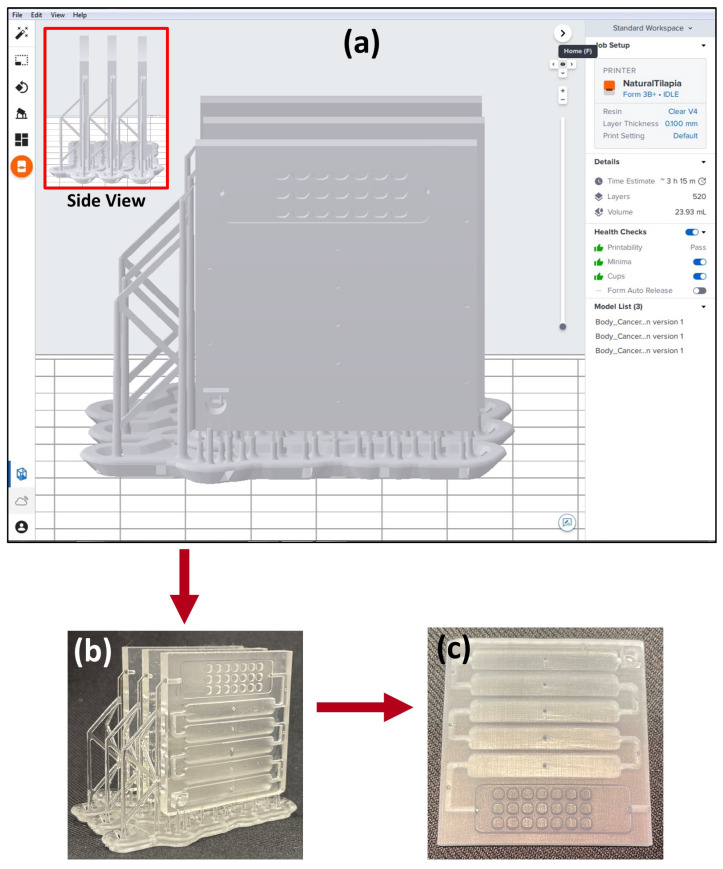
Design features: (**a**) 3D-printed design from PreForm software showing how the supports were generated (inset showing the side view). (**b**) Photos of the 3D-printed device with supports before the post-processing step and (**c**) after post-processing.

The corrected and finalized design is exported from the Fusion 360 software and uploaded to PreForm software that generates appropriate support (Figure 2a,b) for the final design and uploads the design file to the 3D printer. The supports seen in Figure 2a can be customized or can be autogenerated from the PreForm software (version 2.20.0). The supports make sure that the print is not misaligned and minute details are not lost during the print. Typically, multiple devices are printed at once. After printing the devices, they are post-processed.

During post-processing, the supports are cut off from the printed devices, which are then sonicated in 2-propanol to remove residual resin on the exterior and interior of the device. Next, the device is lightly brushed and washed with 2-propanol then cured in an oven at 60 °C for 30 min (Figure 2c).

#### 2.2.2. Biomarker Selection

The choice of target protein analytes for the array is of course a very important issue for a successful biomedical assay. In this example, we are working toward an assay useful for general screening of patient serum for aggressive cancers. Biomarker protein selection here was performed using an extensive literature survey. The cancers to be detected were chosen as prostate, breast, ovarian, colorectal, pancreatic, and lung cancers, which all have sub-types with high prevalence and severity. Then, protein biomarkers that might be used for the diagnosis of each of these cancers were assessed from literature reports that included receiver operating characteristic (ROC) analyses. ROC plots [35] are statistical tools that provide estimates of sensitivity and specificity of diagnostic utility for an assigned task, e.g., detection of a cancer from levels of biomarkers in serum. ROCs are x-y plots of true positive rate (sensitivity) vs. the false positive rate (100% specificity) for sample levels as dependent variables [36]. We chose protein biomarkers that had high sensitivity and specificity (e.g., >70%) in the published ROC curve as suitable biomarkers for our immunoassay. Based on this process, we decided upon PEDF, CA-125, IL-8, CD-14, and Cyr61 as our initial choices of protein biomarker analytes. Another issue that needs to be checked is the availability of a commercial capture and detection antibodies for each protein analyte. Antibodies used should have dissociation constants with target proteins < 6 nM. This is rarely a problem for known protein biomarkers since many thousands of antibodies are commercially available. If a protein analyte does not have commercial antibodies, production can usually be contracted out to a suitable antibody generation service. Two antibodies that bind at different sites are needed for each biomarker protein. Monoclonal antibodies are the best choice, but one monoclonal and one polyclonal antibody will usually work just as well. Aptamers [37] can also be used or aptamer–antibody pairs. Biotinylated detection antibodies for each biomarker (Ab${}_{2}$), and streptavidin poly-horseradish peroxidase (ST-HRP) are also used.

#### 2.2.3. Immunoassay Development

**Figure 3:**
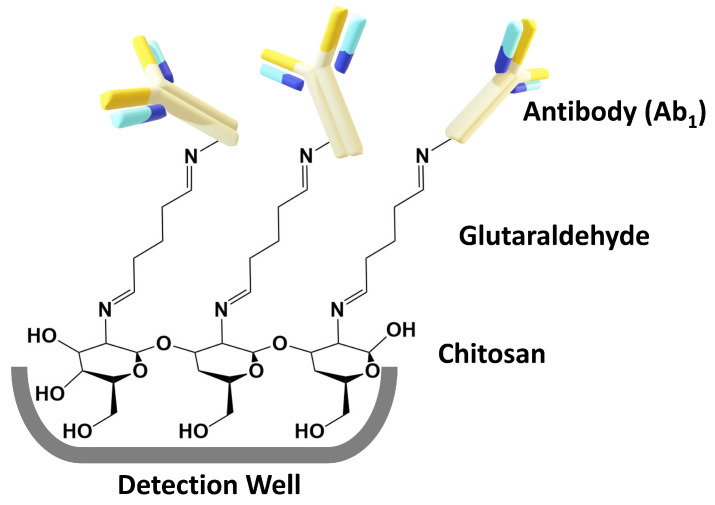
Chemical attachment of Ab${}_{1}$s to chitosan hydrogel in array wells.

Next, we describe the immunoassay development. First, processed arrays are functionalized within each detection well to capture the biomarker proteins (PEDF, CA-125, IL-8, CD-14, and Cyr61) using a primary antibody (Ab${}_{1}$) for each biomarker. Optimized chitosan–glutaraldehyde reactions are used to immobilize each Ab${}_{1}$ in its assigned detection wells [38]. Briefly, chitosan (0.5 mg/mL in 0.05 M HCl, pH 4) is added to the detection wells (5 µL) and incubated for 3 hr, excess is removed (by gently dabbing with Kimwipes), and the device vacuum dried overnight at room temperature. This forms a layer of chitosan hydrogel in each detection well and increases the surface area available to bind Ab${}_{1}$s. This layer is then functionalized by incubating 3% glutaraldehyde in the detection well for 3 h. Gluteraldehyde forms Schiff’s bases with amines on chitosan and leaves free aldehydes available to react with amines on Ab${}_{1}$s (Figure 3). The interaction between chitosan and the detection wells was studied extensively by Sharafeldin et al. [38]. They found that the chitosan film is strongly physisorbed onto the 3D-printed detection wells. This 3D film is a porous hydrogel with 98% water that provides a high surface area for the antibodies to bind.

**Figure 4:**
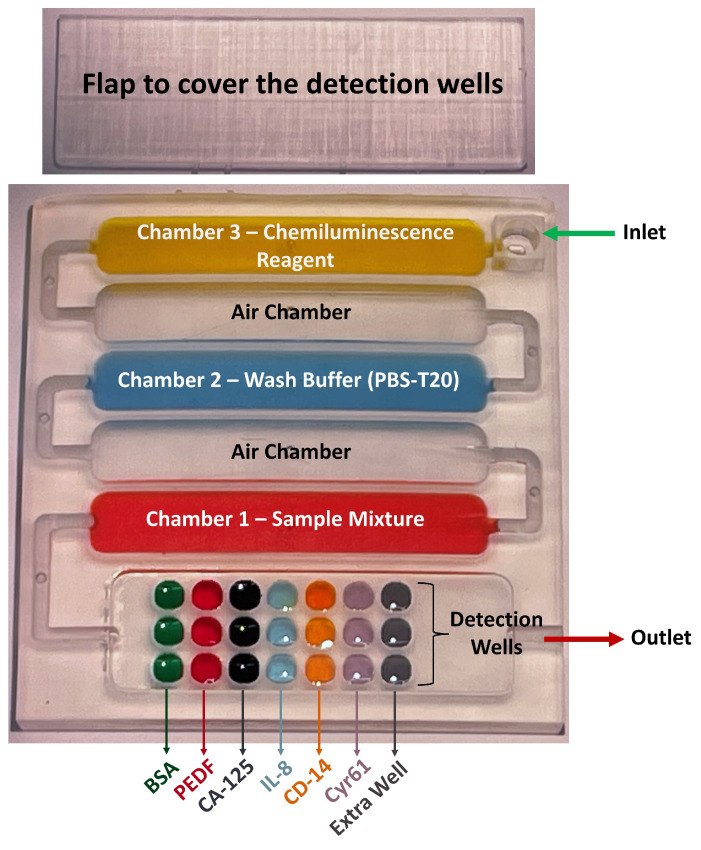
Final version of the 3D-printed microfluidic immunoarray showing what goes in each reagent chamber and what Ab${}_{1}$s are attached in each detection well.

Next, excess glutaraldehyde is removed (by gently dabbing using Kimwipes^®^ (from Kimtech™, Roswell, GA, USA)), and the devices are vacuum dried for 1 h at RT. The vacuum-dried devices are then incubated overnight at 4 °C with the optimized concentration of Ab${}_{1}$, or 1% Bovine Serum Albumin (BSA) as controls, in each assigned detection well, using 3 wells for each biomarker and the control (Figure 4). Excess Ab${}_{1}$ is gently removed using Kimwipes and loosely bound Ab${}_{1}$ are washed away with pH 7.4 PBS + 0.01% Tween-20, (PBS-T20). Then, the detection wells are filled with Blocker Casein (This is sold as a 1× solution) for 1 h and washed with PBS.

**Figure A1:**
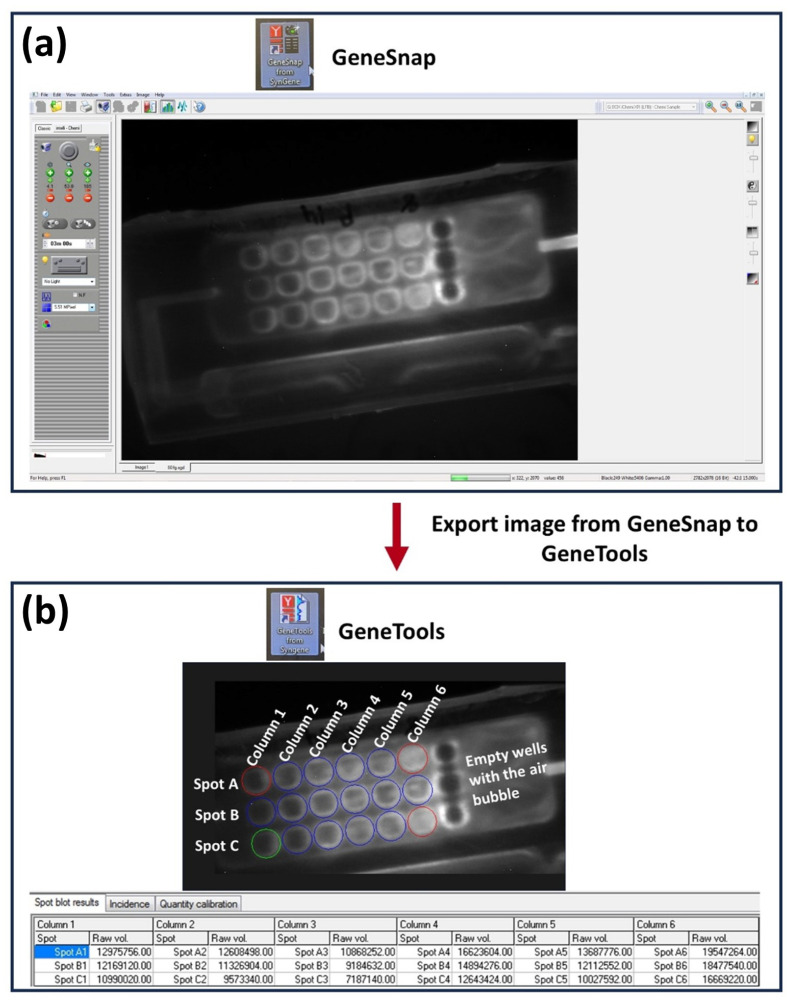
Image processing. (**a**) Image captured by Genesnap being exported to (**b**) GeneTools to obtain a numerical value of the relative CL intensity for each detection well.

**Figure A2:**
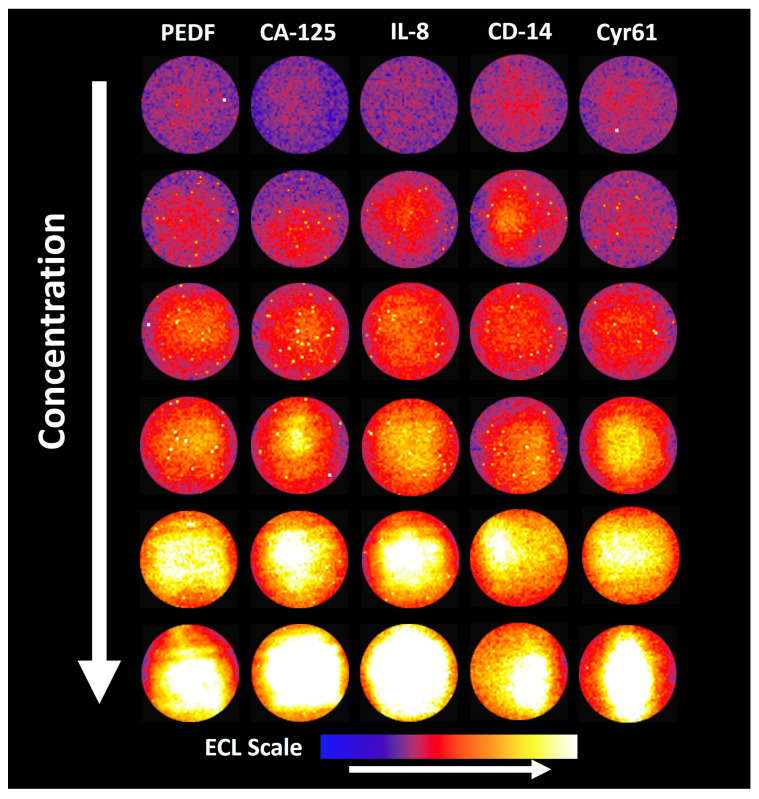
Re-colorized image of the detection wells for increasing protein concentrations for PEDF, CA-125, IL-8, CD-14, and Cyr61.

**Figure A3:**
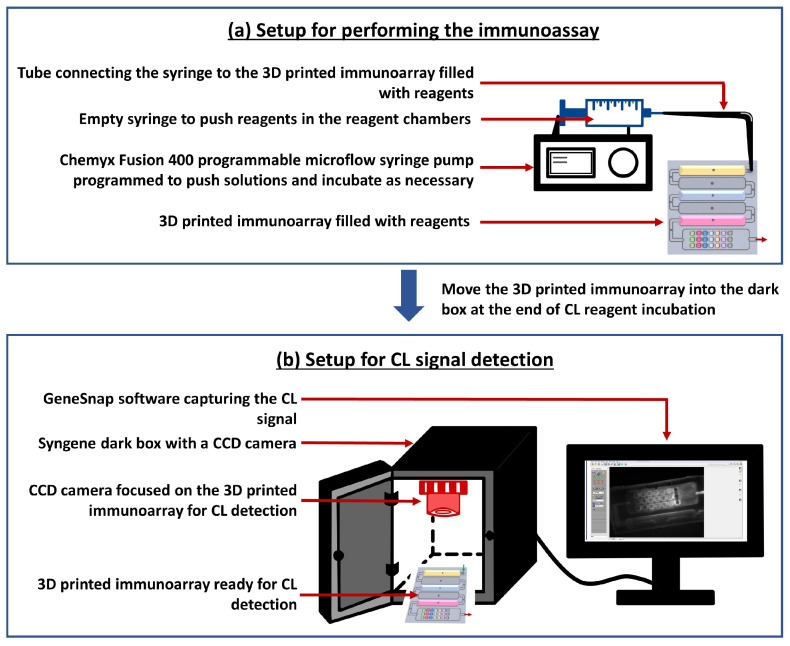
Schematic diagram of the (**a**) experimental setup for the immunoassay and the (**b**) hardware setup for the chemiluminescence (CL) signal capturing.

To prepare for an assay, the Ab${}_{1}$-equipped detection wells are covered with the 3D-printed flap (Figure 4, top) using Loctite waterproof superglue. Then, the reagent chambers are filled with the corresponding solutions (350 µL ea.) using the small hole in the top of each chamber. Reagent chamber 1 (Figure 4) is filled with a reaction mixture that contains sample, biotinylated detection antibodies for each biomarker (Ab${}_{2}$), and streptavidin poly-horseradish peroxidase (ST-HRP). The sample here denotes standard proteins with known concentrations or human serum. At this point, concentrations of Ab${}_{2}$ for each biomarker have been previously optimized (see next section), as is the concentration of ST-HRP [38]. This mixture is incubated in the sample chamber for 5 min to form protein–Ab${}_{2}$–biotin–ST–HRP complexes before passing into the detection chamber. Reagent chamber 2 is filled with PBS-T20 as wash buffer. Reagent chamber 3 is filled with SuperSignal West Femto-luminol Maximum Sensitivity Substrate, which is a 1:1 solution of H${}_{2}$O${}_{2}$ and femto-luminol/enhancer (CL reagent). Next, tape is used to cover reagent chamber holes, a Chemyx Fusion 400 programmable microflow syringe pump is connected to the inlet (Figure 4), and solutions are delivered into the detection wells. This experimental setup is shown in Figure A3a. The pump has a flow rate of 150 µL/s for 20 min stopped-flow incubation of the reaction mixture in the detection wells. Next, sample solution is pumped into detection wells and incubated for 20 min. Then, the PBS-T20 wash buffer pumps through the detection wells, followed by CL reagent into the detection wells with programmed stop-flow incubation for 5 min. At this point, detection is implemented by capturing an integrated image of the detection wells for 180 s using a charge-coupled device (CCD) camera (Syngene G: box F3) in a dark box controlled by a computer with GeneSnap software. This is performed by transferring the immunoarray into the dark box after the detection wells are incubated with the CL reagents. This dark box has a CCD camera that is controlled by the GeneSnap software. Using this software, the detection wells are focused onto the CCD camera, which captures the CL intensity in the form of a grayscale image. The setup of the hardware to capture this image is shown in Figure A3b. This grayscale image is then exported to the GeneTools software. In this software, once the area of the detection well is defined, the software generates a numerical value for the brightness in the detection well against the dark background (Figure A1). This value is used for further analyses. Finally, raw output images from GeneSnap can be re-colorized to produce representative images using Photoshop since the raw images are in grayscale (Figure A2) and converted into a numerical scale that can be used to construct calibration curves.

#### 2.2.4. Optimization Steps

It is important to optimize Ab${}_{2}$ and Ab${}_{1}$ concentrations, sample incubation time, and flowrate before the assay validation studies. Defining the best Ab${}_{2}$ and Ab${}_{1}$ concentrations is critical, since if they are not optimized, sensitivity may be poor or even non-existent. In our procedure, the Ab${}_{2}$ concentration is first optimized for each protein separately. The flow rate is kept low (50 µL/min) for optimization assays but is subjected to a final optimization as well.

**Figure 5:**
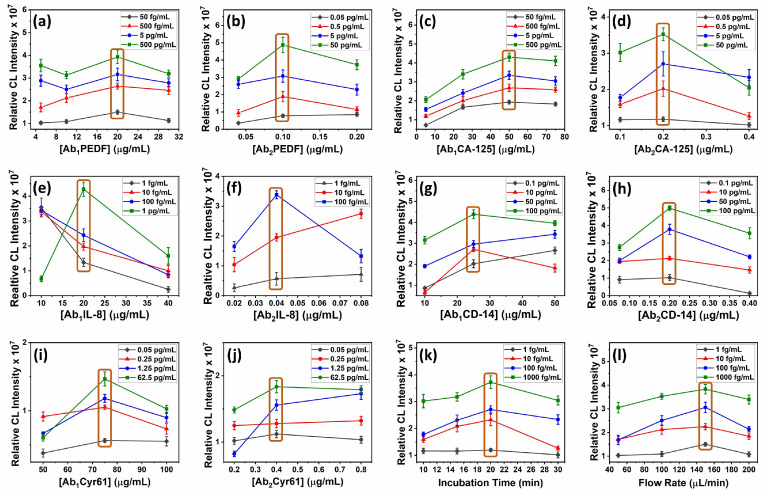
Plots for Ab${}_{2}$ and Ab${}_{1}$ optimization of (**a**,**b**) PEDF, (**c**,**d**) CA-125, (**e**,**f**) IL-8, (**g**,**h**) CD-14, and (**i**,**j**) Cyr61 and plots for (**k**) incubation time and (**l**) flow-rate optimizations, with inset data showing analyte concentrations. The orange-colored boxes are located at optimum concentrations where signal increase per unit concentration is largest.

For Ab${}_{2}$ optimization, the array device is loaded with a ‘maximum Ab${}_{1}$ concentration’. This is to ensure that we are providing the conditions necessary for a maximum range of analyte concentrations to be captured. Approximately 5× the Ab${}_{1}$ concentration recommended by the DuoSet ELISA kits (used in this assay as the source of Ab${}_{1}$, Ab${}_{2}$, and the analyte: refer to Materials) is considered as the ‘maximum Ab${}_{1}$ concentration’. These ‘maximum Ab${}_{1}$ concentrations’ are [Ab${}_{1}$PEDF] = 20 µg/mL, [Ab${}_{1}$CA-125] = 50 µg/mL, [Ab${}_{1}$IL-8] = 20 µg/mL, [Ab${}_{1}$CD-14] = 50 µg/mL, and [Ab${}_{1}$Cyr 61] = 75 µg/mL). Then, the immunoassay is performed at varying Ab${}_{2}$ concentrations ([Ab${}_{2}$PEDF] = 0.04, 0.1, and 0.2 µg/mL, [Ab${}_{2}$CA-125] = 0.1, 0.2, and 0.4 µg/mL, [Ab${}_{2}$IL-8] = 0.02, 0.04, and 0.08 µg/mL, [Ab${}_{2}$CD-14] = 0.075, 0.2, and 0.4 µg/mL, and [Ab${}_{2}$Cyr 61] = 0.2, 0.4, and 0.8 µg/mL) across different concentrations of analyte proteins that encompass the approximate desired dynamic range for each ([PEDF] = 0.05, 0.5, 5, and 50 pg/mL, [CA125] = 0.05, 0.5, 5, and 50 pg/mL, [IL-8] = 1, 10, 100, and 1000 fg/mL, [CD-14] = 0.1, 10, 50, and 100 pg/mL, and [Cyr61] = 0.05, 0.25, 1.25, and 62.5 pg/mL ). The Ab${}_{2}$ concentration that gives the best signal separation in triplicate experiments between the 4 protein concentrations in ascending order (Figure 5) is chosen as the optimized Ab${}_{2}$ concentration. Samples are known concentrations of biomarker + Ab${}_{2}$ for each biomarker + ST-HRP in PBS and incubated in detection chambers for 30 min.

Using the optimized Ab${}_{2}$ concentration, the immunoassay is now performed to optimize Ab${}_{1}$ ([Ab${}_{1}$PEDF] = 5, 10, 20, and 30 µg/mL, [Ab${}_{1}$CA-125] = 5, 25, 50, and 75 µg/mL, [Ab${}_{1}$IL-8] = 10, 20, and 40 µg/mL, [Ab${}_{1}$CD-14] = 10, 25, and 50 µg/mL, and [Ab${}_{1}$Cyr 61] = 25, 50, 75, and 100 µg/mL) across multiple protein concentrations ([PEDF] = 0.05, 0.5, 5, and 50 pg/mL, [CA125] = 0.05, 0.5, 5, and 50 pg/mL, [IL-8] = 1, 10, 100, and 1000 fg/mL, [CD-14] = 0.1, 10, 50, and 100 pg/mL, and [Cyr61] = 10, 50, 250, and 500 fg/mL ), and the optimum Ab${}_{1}$ concentration is determined as above. This protocol has served us well for signal optimization of many microfluidic assays [18,21,22,23,24,25,26,38,39].

As the last step, incubation time and flow rate are optimized here using IL-8 because IL-8 is the biomarker with the lowest normal serum level (Table A1). The immunoassay is performed across multiple protein concentrations ([IL-8] = 1, 10, 100, and 1000 fg/mL) using optimized Ab${}_{1}$ and Ab${}_{2}$ concentrations and varying sample incubation times (10, 15, 20, and 30 min). The time that gives the best signal separation was used as the optimum sample incubation time. The sample solution for this optimization step is a mixture of the protein biomarker, Ab${}_{2}$, and ST-HRP in PBS. Flow rate is optimized by varying the flow rate (50, 100, 150, and 200 µL/min), repeating the above assay, and choosing the flow rate that gives best sensitivity and LOD.

#### 2.2.5. Assay Validation

Assay validation is performed by obtaining calibration plots and performing spike-recovery studies. Calibration plots are obtained by performing an optimized immunoassay for a range of protein analyte concentrations. The sample solution is a mixture of every biomarker, every Ab${}_{2}$ at the optimized concentration, and ST-HRP in 1% calf serum. The analyte concentration range used in this case was 0.01–1500 fg/mL. The CL intensities from these immunoassays are plotted against concentration from which the linear dynamic range and the limit of detection (LOD, 3xSD of the blank above the blank signal) are obtained for each protein.

Spike-recovery studies are conducted to validate accuracy of the assay by spiking pooled human serum with known concentrations (15, 75, 200, and 500 fg/mL) of each analyte and assaying these. Then, calibration plots are used to determine the concentration of each analyte. Using the spiked concentration and the found concentration from the calibration curve, the % spike recovery is calculated.

## 3. Results

### 3.1. Biomarker Selection

As mentioned earlier, a comprehensive literature search was conducted on 48 potential protein biomarkers associated with prostate, breast, ovarian, colorectal, pancreatic, and lung cancers [40,41,42,43,44,45,46,47,48,49,50]. The evaluation encompassed an assessment of these biomarkers in terms of their expression levels in both healthy individuals and cancer patients, as well as their sensitivity and specificity indicated by the literature ROC plots based on patient data. From this analysis, 11 possible biomarker candidates were identified with clinical sensitivity and selectivity thresholds for identifying cancers vs. no cancer at >70% (Table A1). Out of these biomarkers, five were used in the multiplexed array development study presented here.

### 3.2. Designing and 3D Printing the Array

Before designing the 3D-printed device, we defined microchannel dimensions, shape, configuration of the microchannels and the associated functionalization areas (reagent chambers and detection wells), detection method, number of reagents needed to perform the assay, material, and printer used for 3D printing and multiplexing. A previous microchannel/reagent chamber we developed for chemiluminescence detection of three proteins in duplicate was designed with eight wells connected by cylindrical flow lines. However, in the present array, we targeted a minimum of five proteins determined in triplicate, with three controls and three overflow wells. We found that the linear flow line well arrangement did not fill wells equally, especially for the later wells in the line. We thus decided on a flat, open well arrangement much like the well’s arranged in a 96-well plate. All the wells have open tops on the same plane, and the incoming solution flows over them in a solution plane to fill all the wells equally (Figure 2c). Dimensions of the reagent chambers were adjusted to hold enough reagent to fill all the detection wells sequentially, and rounded edges facilitate smooth solution flow without bubbles or retaining solution in corners. Air chambers were included between reagent chambers to create air barriers to prevent reagent mixing during flow. Detection wells were designed with a rounded shape, and wells can be included to hold either 5 µL or 10 µL each. We used three detection wells per biomarker and three for the control for multiplexing purposes. In our optimal design, the first three vertical wells were the control, and the remainder included three wells per biomarker and three end wells to capture overflow.

Detection was by chemiluminescence; therefore, a clear resin was used to 3D print the device so that the CCD camera can capture a clear image of the detection wells. This resin was compatible with the choice of 3D printer used as well. A Formlabs Form 3 printer was used.

**Figure 6:**
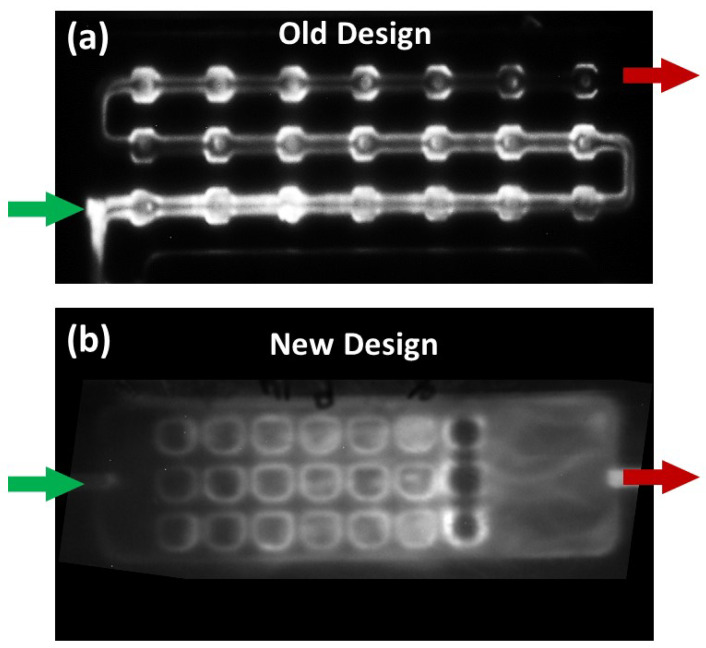
Image of (**a**) closed well design with uneven signal distribution and (**b**) open well design with uniform signal distribution where the green arrow and the red arrow indicates the direction of the inlet and the outlet respectively.

While we went through several array designs, the final version of the array was designed with open wells (Figure 2 and Figure 4). In this version, solutions did not flow through the detection wells in a linear sequence, rather they filled the detection wells by flowing solution over the top. Since the solutions were no longer flowing from one detection well to another, the signal distribution was uniform (Figure 6). This is clearly seen in the SI video (for demonstration, the device used was printed with only one reagent chamber). The reagents can be seen to fill the detection wells uniformly by solution flowing over their tops with minimal disturbing of their immobilized contents. We also found out that protein analyte (or sample), Ab${}_{2}$-biotin, and ST-HRP can be premixed without compromising assay performance. Therefore, our final version has fewer reagent chambers and a separate flap to cover detection wells.

Dimensions of the final array were 45 mm × 43 mm × 4 mm, and the flap was 44 mm × 14 mm (Figure 4). The device consists of six parallel chambers (350 µL each) and connecting channels to allow sequential flow of reagents. There are micro-holes over the first five chambers and connecting channels. The first, third, and the fifth are reagent chambers, and second and fourth are air chambers. The inlet is connected to the first chamber and the outlet to the last chamber. The inlet connects to an automated syringe pump via a flexible tube. The sixth chamber contains the detection wells. There are 21 detection wells (2.5 mm × 2.5 mm × 1 mm) aligned in a 3 × 7 configuration with 3 wells per biomarker. There is an extra row of detection wells at the outlet end to trap overflow.

### 3.3. Optimization Steps

**Figure 7:**
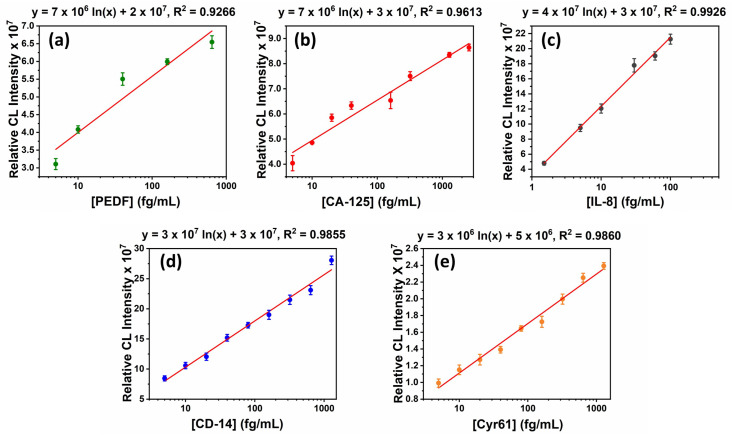
Calibration plots (n = 3) for (**a**) PEDF, (**b**) CA-125, (**c**) IL-8, (**d**) CD-14, and (**e**) Cyr61.

Each biomarker had a different optimum pair (Ab${}_{2}$ and Ab${}_{1}$) of antibody concentrations that yielded the best signal separation across the selected protein concentrations (Table A2). The optimized incubation time was 20 min and the best flow rate 150 µL/min (Figure 5k,l). All of these optimizations were used to construct the calibration plots (Figure 7a–e).

As shown in Figure 5, most of the optimizations were performed by using three variants of the parameter being optimized (x-axis). However, there were few where four variants were considered. The fourth variant had to be included when the third variant gave the best signal separation. To check whether the third variant is the best option, a fourth had to be tested. For example, in Ab${}_{1}$ optimization for PEDF, initially, 5, 10, and 20 µg/mL of Ab${}_{1}$PEDF were tested. Since this experiment concluded that the highest considered concentration (20 µg/mL) was the optimum, one higher concentration had to be tested to confirm that the increasing trend seen on the plot (Figure 5a) does not continue resulting in another higher concentration being the optimum. In the case of flow-rate optimization, 100 µL/min could have been selected instead of 150 µL/min. However, 100 µL/min significantly increased the assay time. Therefore, 150 µL/min was taken as the optimum flow rate.

### 3.4. Assay Validation

The calibration experiments performed using the optimized parameters yielded different linear dynamic ranges and limits of detections for each biomarker as shown in Figure 7. The table also summarizes the linearity of the calibration plots as well as the calibration plot equations. CD-14 gave the lowest LOD at 0.01 fg/mL, and the highest linear range was from 0.01 fg/mL–100 pg/mL. The best linearity was seen for IL-8, yet it had the lowest linear range.

The spike-recovery studies yielded the following % recoveries for each biomarker at each analyte concentration used for the experiment (Table 1). All the spike-recovery values are within 100 ± 20%.

## 4. Discussion

The design of a 3D-printed microfluidic device for an immunoassay is a complicated, overlapping multistep process. Nonetheless, with ambit of experience and rational planning, it is a cheap and versatile approach to develop a 3D-printed, multiplexed immunarray. Thus, many research groups and industries are using 3D printers in many types of microfluidic arrays [51], e.g., for detecting many types of cancer biomarkers [17,39,52,53], COVID-19 [26,54], for IgEs [55], and detecting inflammation biomarkers [56,57]. The assay presented in this study was designed to detect five protein biomarkers concurrently (multiplexed) for multiple cancers, but the approaches can be similar for other types of multiplexed assays.

The first step in designing a 3D-printed microfluidic device is to have a rough idea of how the assay will be achieved, including reagents that will be used, number of analytes to be detected, detection technique, budget, and reagent availability. Then, the device is designed according to these parameters and the desired sensitivity, reproducibility, detection limits, and dynamic concentration range. There are several software choices available for designing microfluidic devices for 3D printing [58,59,60]. We used Autodesk Fusion 360 as our preference. The 3D design went through multiple edits where the orientation and dimensions of the reagent chambers, air chambers, and detection wells were changed (Figure 1). These changes were a result of trial and error in running test assays. For example, a closed well design with linear detection scheme (Figure 6a) was changed to an open well design similar to a standard well plate because the closed wells did not fill uniformly when there were too many connected, resulting in an uneven signal distribution (Figure 6). Open wells filled uniformly and also made the functionalization of the detection wells with antibodies easier. The reagents were no longer flowing through one detection well to the next. Rather, the wells were filled by the solution entering from the top of the “well plate” uniformly (Video S1). This change in the detection wells was also required in designing a flap to cover the wells to perform the assay (Figure 1 and Figure 4). In addition, an extra set of three detection wells that were empty with no antibodies were included next to the outlet as overflow wells that ensured proper filling of the preceding wells. This avoided air bubbles forming over the upstream detection wells. The orientation of reagent chambers was also changed multiple times to obtain an optimum flow of reagents. Openings were introduced above each air chamber and the reagent chamber for reagent filling. These openings served both as entry points for pipetting reagents in and as vents to prevent back pressure during reagent filling.

The reagents filled into the three reagent chambers were the sample, the PBS-T20 wash buffer, and the CL reagent. The sample solution was a mixture of several constituents. Namely, the biomarker, biotinylated Ab${}_{2}$ for each biomarker, and ST-HRP. In contrast, these components are introduced to the detection wells sequentially in a traditional immunoassay [61]. In this mixture, the biotinylated Ab${}_{2}$ makes a complex with its specific biomarker and the ST-HRP (Ag-Ab${}_{2}$-HRP). This complex starts forming during the 5 min incubation of the mixture in the reagent chamber and the 20 min incubation in the detection chamber. In addition, during this 20 min incubation, the Ag binds to the Ab${}_{1}$ immobilized on the detection well. This amount of Ab${}_{1}$ that is immobilized in each well and the amount of Ab${}_{2}$ and ST-HRP added to the sample were optimized to achieve the best signal-to-noise ratio. The flowrate and the incubation times were optimized as well by varying the parameter to be optimized across a range of analyte concentrations and keeping all the other parameters a constant. The value of the variant that gave the best signal separation in the increasing order of analyte concentration was selected as the optimized value (Figure 5). The order of the optimizations was also important.

First, the Ab${}_{2}$ concentration was optimized. During this step, the detection wells were saturated with a ‘maximum Ab${}_{1}$ concentration’ to ensure maximum protein capture. This guarantees that the optimum [Ab${}_{2}$] is not restricted by having a limited amount of protein in the detection well. The ‘maximum Ab${}_{1}$ concentration’ was achieved by immobilizing at least five times the amount recommended by the corresponding ELISA kit. Second, the Ab${}_{1}$, then the incubation time, and finally the flow rate were optimized. For proceeding optimizations, previously optimized values were used. These optimizations are very important in obtaining the best sensitivity, specificity, accuracy, linearity/LOD, and robustness [62].

The calibration experiments provide a quantitative assessment of assay performance by portraying the linear dynamic ranges and limits of detection for each biomarker. This information is critical for understanding the assay’s sensitivity and dynamic range [62]. The observation of varying linear dynamic ranges and LOD among different biomarkers points out the uniqueness of each biomolecular interaction under investigation. The inclusion of calibration plot equations facilitates the translation of raw data into quantifiable results, promoting transparency and ease of implementation. For the present application, as desired, all the calibration plots are within a range that can be used to measure normal and cancer patient levels when the serum is diluted (Table A3).

Spike-recovery studies investigate the assay’s accuracy by testing its ability to accurately recover known concentrations of biomarkers in liquid media similar to that used for real samples. All percent recovery values here were within the analytically acceptable range of ±20% [63]. Higher concentrations were recovered in certain instances due to random error and non-specific binding. However, all the recoveries are within the analytically acceptable range as stated above.

## 5. Conclusions

By explaining rigorous design, optimization, and validation components, this paper provides a holistic understanding of the steps involved in designing a 3D-printed microfluidic immunoarray for ultrasensitive multiple protein detection. Designs, analytical parameters, calibrations, and accuracy often need to be optimized in an iterative manner. We hope our descriptions above will serve as a foundation for future applications in biomedical and other 3D-printed analysis systems. In addition, further exploration of the relationships between design parameters, optimization outcomes, and assay validation could lead to deeper insights and enhancements in similar devices and methodologies.

## Figures and Tables

**Table 1:** Spike-recovery data for each biomarker.

		Spiked Conc: (fg/mL)			
		15	75	200	500
PEDF	Found Concentration (fg/mL)	17 (±8)	66 (±4)	177 (±19)	580 (±31)
	Percent Recovery (%)	111 (±10)	88 (±13)	89 (±16)	116 (±14)
CA-125	Found Concentration (fg/mL)	14 (±2)	83 (±11)	190 (±15)	522 (±25)
	Percent Recovery (%)	92 (±6)	111 (±14)	95 (±7)	104 (±11)
IL-8	Found Concentration (fg/mL)	17 (±5)	65 (±8)	169 (±10)	569 (±24)
	Percent Recovery (%)	110 (±14)	86 (±8)	84 (±16)	114 (±17)
CD-14	Found Concentration (fg/mL)	17 (±3)	63 (±4)	164 (±12)	470 (±18)
	Percent Recovery (%)	115 (±15)	84 (±9)	82 (±13)	94 (±12)
Cyr61	Found Concentration (fg/mL)	16 (±5)	86 (±15)	211 (±15)	586 (±21)
	Percent Recovery (%)	106 (±11)	115 (±13)	106 (±19)	117 (±14)

## Data Availability

The data that support the findings of this study are available on request from the corresponding author, [J.F.R.].

## Supplementary Materials

The following supporting information can be downloaded at: https://www.mdpi.com/article/10.3390/mi14122187/s1. Video S1: Displaying the process of filling detection wells in the ‘open well’ 3D printed microfluidic device.

## Abbreviations

The following abbreviations are used in this manuscript:

LOD	Limit of Detection
3D	Three Dimensional
CL	Chemiluminescence
PEDF	Human Pigment Epithelium-Derived Factor
CA-125	Human Cancer Antigen 125
IL-8	Human Interleukin-8
CD-14	Human Cluster of Differentiation 14
Cyr61	Human Cysteine-rich Angiogenic Inducer 61
BSA	Bovine Serum Albumin
CCD	Charge Coupled Device
PBS	Phosphate Buffer Saline
PBS-T20	Phosphate Buffer Saline—Tween 20
CFD	Computational Fluid Dynamic
ROC	Receiver Operating Characteristic
Ab${}_{2}$	Biotinylated Detection/Secondary Antibody
ST-HRP	Streptavidin poly-Horseradish Peroxidase
Ab${}_{1}$	Capture/Primary Antibody
RT	Room Temperature
SD	Standard Deviation

## Appendix A

**Table A1:** Summary of protein biomarkers targeting various cancers with sensitivity and selectivity higher than 70% and AUC close to 1, identified as good biomarker choices to be included in the multiplexed protein assay for the development of a global cancer blood test. (**${}^{1}$** non-small cell lung cancer, **${}^{2}$** small cell lung cancer).

Biomarker	Cancer	Normal Con.	Conc. in Cancer Patients	Sensitivity (%)	Specificity (%)	AUC
**PTHrp**	Breast, prostate	<2.5 pM	>2.5 pM	100	80.0	0.96
CA 15-3	Breast	<30 U/mL	>30 U/mL	77.5	85.0	0.89
CA-125	Ovarian	<35 U/mL	>35 U/mL	79.0	82.0	0.87
IGFBP-4	Ovarian	400.9 ng/mL	1344.09 ng/mL	73.0	93.0	0.82
IL-8	Colorectal	2.38–20.05 pg/mL	0.61–32.86 pg/mL	70.0	91.0	0.92
sDC-SIGN & sDC-SIGNR	Colorectal	-	-	98.7	98.8	0.99
Cyr61	Colorectal	<92.0 pg/mL	>92.0 pg/mL	83.0	97.0	0.94
CEACAM1	Lung (NSCLC ${}^{1}$)	226.80–490.11 ng/mL	381.30–968.13 ng/mL	97.0	82.0	0.96
ProGRP	Lung (SCLC ${}^{2}$)	<50 pg/mL	>50 pg/mL	84.0	95.0	0.72
PEDF	Breast, prostate	12.93 µg/mL	<9 µg/mL	64.5	70.6	0.70
CD-14	Breast, prostate	4.89 µg/mL	6.69 µg/mL	58.9	82.3	0.73

**Table A2:** Optimum Ab_1_ and Ab_2_ concentrations for each biomarker.

Biomarker	Optimum Ab_1_ (µg/mL)	Optimum Ab_2_ (µg/mL)
PEDF	20	0.1
CA-125	50	0.2
IL-8	20	0.04
CD-14	25	0.2
Cyr61	75	0.4

**Table A3:** Comparison of the linear range of the calibration plots with the normal and cancer patient levels of each biomarker.

Biomarker	Linear Range	Limit of Detection (fg/mL)	Normal Conc. in Serum	Conc. in Cancer Patient Serum
PEDF	5–625 fg/mL	3	12. 93 µg/mL	<9 µg/mL
CA-125	5 fg/mL–15.6 pg/mL	1.5	<35 U/mL	>35 U/mL
IL-8	1.5–100 fg/mL	1.5	2.38–20.05 pg/mL	0.61–32.86 pg/mL
CD-14	0.01 fg/mL–100 pg/mL	0.01	4.89 µg/mL	6.69 µg/mL
Cyr61	5–500 fg/mL	4	<92.0 pg/mL	>92.0 pg/mL
